# A shorter post-exposure prophylaxis regimen for rabies, Pakistan

**DOI:** 10.2471/BLT.20.275453

**Published:** 2021-04-29

**Authors:** Naseem Salahuddin, Nadia Ansari, Muhammad Aftab Gohar

**Affiliations:** a The Indus Hospital & Health Network, Plot C-76, Sector 31/5, Korangi Crossing, Karachi, 75190, Pakistan.

## Abstract

**Objective:**

To assess the cost and effectiveness of the two-site, 1-week, intradermal rabies post-exposure prophylaxis regimen recommended by the World Health Organization (WHO) in 2018.

**Methods:**

We compared the number of rabies vaccine and rabies immunoglobulin ampoules consumed at The Indus Hospital in Karachi, Pakistan and their cost before and after implementing WHO’s 2018 recommendations. In 2017, patients with suspected rabies-infected bites were treated using the two-site, 4-week, Thai Red Cross regimen, which involved administering four rabies vaccine doses intradermally over 4 weeks and infiltrating immunoglobulin into serious wounds, with the remainder injected into a distant muscle. In 2018, patients received three vaccine doses intradermally over 1 week, with a calculated amount of immunoglobulin infiltrated into wounds only. Remaining immunoglobulin was saved for other patients. The survival of patients bitten by apparently rabid dogs was used as a surrogate for effectiveness.

**Findings:**

Despite treating 8.5% more patients in 2018 (5370 patients) than 2017 (4948 patients), 140 fewer ampoules of rabies vaccine and 436 fewer ampoules of rabies immunoglobulin were used, at a cost saving of 4202 United States dollars. Of 56 patients bitten by apparently rabid dogs, 50 were alive at 6-month follow-up. The remaining six patients could not be contacted but did not present to any hospital with rabies.

**Conclusion:**

The new regimen was more economical than the two-site, 4-week regimen and was equally effective. This regimen is recommended for preventing rabies in countries where the disease is endemic and rabies vaccine and immunoglobulin are in short supply.

## Résumé

**Objectif:**

Évaluer le coût et l'efficacité du schéma de prophylaxie post-exposition à la rage par voie intradermique en deux sites sur une semaine, tel que recommandé par l'Organisation mondiale de la Santé (OMS) en 2018.

**Méthodes:**

Nous avons comparé le nombre d'ampoules de vaccin antirabique et d'immunoglobuline antirabique consommées à l'Indus Hospital à Karachi, au Pakistan, ainsi que leur coût avant et après l'application des recommandations émises en 2018 par l'OMS. En 2017, les patients présentant une morsure avec suspicion de rage étaient traités selon le schéma de la Croix-Rouge thaïlandaise d'injection en deux sites sur quatre semaines, ce qui impliquait l'administration intradermique de quatre doses de vaccin en l'espace de quatre semaines et l'infiltration d'immunoglobuline dans les blessures graves, avec injection du reste dans un muscle distant. En 2018, les patients ont reçu trois doses par injection intradermique en l'espace d'une semaine, avec une quantité précise d'immunoglobuline infiltrée uniquement dans les blessures. Le reste de l'immunoglobuline était conservé pour d'autres patients. La survie des patients mordus par des chiens potentiellement enragés a servi d'indicateur d'efficacité.

**Résultats:**

Malgré une hausse de 8,5% des patients traités en 2018 (5370 patients) par rapport à 2017 (4948 patients), l'hôpital a utilisé 140 ampoules de vaccin antirabique et 436 ampoules d'immunoglobuline antirabique en moins, économisant ainsi 4202 dollars américains. Sur les 56 patients mordus par des chiens potentiellement enragés, 50 étaient toujours vivants 6 mois plus tard. Les six patients restants n'ont pas pu être contactés, mais aucun ne s'est rendu à l'hôpital pour être traité contre la rage.

**Conclusion:**

Le nouveau schéma s'est révélé moins onéreux que le schéma d'injection en deux sites sur quatre semaines, tout en affichant le même niveau d'efficacité. Il est donc recommandé pour lutter contre la rage dans les pays où la maladie est endémique, mais aussi où les vaccins antirabiques et l'immunoglobuline antirabique se font rares.

## Resumen

**Objetivo:**

Evaluar el coste y la eficacia de la pauta de profilaxis postexposición a la rabia intradérmica de dos sitios, con una semana de duración, según recomiendó la Organización Mundial de la Salud (OMS) en 2018.

**Métodos:**

Se comparó el número de vacunas para la rabia y de ampollas de inmunoglobulina antirrábica utilizadas en el Hospital Indus de Karachi (Pakistán) y su coste antes y después de aplicar las recomendaciones de la OMS de 2018. En 2017, los pacientes con mordeduras presuntamente infectadas por la rabia fueron tratados con el régimen de la Cruz Roja tailandesa de dos sitios y 4 semanas, que implicaba la administración de cuatro dosis de vacuna antirrábica por vía intradérmica durante 4 semanas y la infiltración de inmunoglobulina en las heridas graves, y el resto se inyectaba en un músculo distante. En 2018, los pacientes recibieron tres dosis de vacuna por vía intradérmica durante 1 semana, con una cantidad calculada de inmunoglobulina infiltrada solo en las heridas. La inmunoglobulina restante se guardó para otros pacientes. La supervivencia de los pacientes mordidos por perros aparentemente con la rabia se utilizó como sustituto de la eficacia.

**Resultados:**

A pesar de tratar un 8,5% más de pacientes en 2018 (5370 pacientes) que en 2017 (4948 pacientes), se utilizaron 140 ampollas menos de vacuna antirrábica y 436 ampollas menos de inmunoglobulina antirrábica, con un ahorro de costes de 4202 dólares estadounidenses. De 56 pacientes mordidos por perros aparentemente con la rabia, 50 estaban vivos a los 6 meses de seguimiento. Los seis pacientes restantes no pudieron ser contactados, pero no se presentaron en ningún hospital con rabia.

**Conclusión:**

El nuevo régimen ha demostrado ser menos costoso que el régimen de inyecciones de dos sitios y cuatro semanas, mientras que proporciona el mismo nivel de eficacia. Por lo tanto, se recomienda para el control de la rabia en los países donde la enfermedad es endémica, pero también donde las vacunas antirrábicas y la inmunoglobulina antirrábica son escasas.

## ملخص

الغرض تقييم تكلفة وفعالية نظام العلاج الوقائي بعد الإصابة بداء الكلب داخل الأدمة في موقعين، ولمدة أسبوع واحد، والذي تم التوصية به من جانب منظمة الصحة العالمية (WHO) في عام 2018.

الطريقة قمنا بمقارنة عدد لقاحات داء الكلب، وأمبولات الجلوبيولين المناعي لداء الكلب، المستهلكة في مستشفى إندوس في كراتشي، بباكستان وتكلفتها قبل تنفيذ توصيات منظمة الصحة العالمية (WHO) لعام 2018، وما بعدها. في عام 2017، تم علاج المرضى المشتبه في تعرضهم للعقر وإصابتهم بداء الكلب، باستخدام نظام الصليب الأحمر التايلنديالتايلاندي عبر موقعين، لمدة 4 أسابيع، والذي تضمن إعطاء أربع جرعات من لقاح داء الكلب داخل الأدمة على مدى 4 أسابيع، وتسريب الجلوبيولين المناعي إلى الجروح الخطيرة، مع حقن الباقي في عضلة بعيدة. في عام 2018، حصل المرضى على ثلاث جرعات من اللقاح داخل الأدمة على مدى أسبوع واحد، مع كمية محسوبة من الجلوبيولين المناعي تم تسريبها إلى الجروح فقط. تم توفير الجلوبيولين المناعي المتبقي للمرضى الآخرين. تم توظيف تعافي المرضى الذين عضتهم كلاب مصابة بالسُعار كبديل للفعالية.

النتائج على الرغم من معالجة مزيد من المرضى بنسبة 8.5% من المرضى في عام 2018 (5370 مريضًا) مقارنة بعام 2017 (4948 مريضًا)، تم استخدام 140 أمبولاً أقل من لقاح داء الكلب، و436 أمبولاً أقل من الجلوبيولين المناعي لداء الكلب، بتوفير في التكلفة بقيمة 4202 دولارًا أمريكيًا. من بين 56 مريضًا عضتهم كلاب مصابة بالسُعار بشكل واضح، كان 50 مريضًا على قيد الحياة في متابعة لمدة 6 أشهر. تعذّر الاتصال بالمرضى الستة الباقين لكنهم لم يقصدوا أي مستشفى بسبب الإصابة بداء الكلب.

الاستنتاج كان النظام الجديد أكثر توفيرًا من النظام ثنائي الموقع لمدة 4 أسابيع، وكان بنفس القدر من الفعالية. يوصى بهذا النظام للوقاية من داء الكلب في الدول التي ينتشر فيها هذا المرض كوباء، ويوجد بها نقص في لقاح داء الكلب والجلوبيولين المناعي.

## 摘要

**目的:**

旨在评估世界卫生组织 (WHO) 在 2018 年推荐的为期 1 周的双部位皮内注射狂犬病暴露后预防方案的成本和有效性。

**方法:**

我们比较了在实施世卫组织 2018 年建议之前和之后，巴基斯坦卡拉奇印度河医院 (Indus Hospital) 消耗的狂犬病疫苗和狂犬病免疫球蛋白安瓶数量及其成本。2017 年，对疑似狂犬病感染的咬伤患者使用泰国红十字会 (Thai Red Cross) 提出的为期 4 周的双部位治疗方案，其中包括在 4 周时间皮内注射四剂狂犬病疫苗，并在严重伤口处浸润注射免疫球蛋白，将其余免疫球蛋白注射到远端肌肉。2018 年，患者在 1 周内接受三剂皮内注射疫苗，仅在伤口处浸润注射一定量的免疫球蛋白，将其余免疫球蛋白留给其他患者。我们将被明显患有狂犬病的狗咬伤的患者的存活时间作为疗效的衡量指标。

**结果:**

虽然 2018 年治疗的患者数量（5370 例）比 2017 年（4948 例）增加了 8.5%，但是狂犬病疫苗使用量减少了 140 安瓶，狂犬病免疫球蛋白使用量减少了 436 安瓶，共节省了 4202 美元。在 56 名被明显患有狂犬病的狗咬伤的患者中，有 50 名在 6 个月的随访中存活。我们未能联系到其余 6 名患者，但他们未曾到任何一家医院看诊狂犬病。

**结论:**

新方案比为期 4 周的双部位预防方案更经济，且二者效果等同。在狂犬病流行，且狂犬病疫苗与免疫球蛋白供应不足的国家，推荐使用此方案预防狂犬病。

## Резюме

**Цель:**

Оценить стоимость и эффективность схемы постконтактной внутрикожной профилактики бешенства с введением вакцины в 2 участка тела в течение 1 недели, рекомендованной Всемирной организацией здравоохранения (ВОЗ) в 2018 году.

**Методы:**

Авторы сравнили количество вакцины против бешенства и ампул антирабического иммуноглобулина, потребленных в больнице Инд в Карачи (Пакистан), и их стоимость до и после выполнения рекомендаций ВОЗ от 2018 года. В 2017 году пациенты с подозрением на укусы, инфицированные бешенством, получали лечение по схеме Красного Креста Таиланда в течение 4 недель с введением вакцины в 2 участка тела, которая включала введение четырех доз вакцины против бешенства внутрикожно в течение 4 недель и инфильтрацию иммуноглобулина в тяжелые раны, а оставшуюся часть вводили внутримышечно в отдаленные от пораженного участка области. В 2018 году пациенты получили три дозы вакцины внутрикожно в течение 1 недели, при этом в раны вводилось только расчетное количество иммуноглобулина. Оставшийся иммуноглобулин был сохранен для других пациентов. Выживаемость пациентов, укушенных явно бешеными собаками, использовалась в качестве суррогатных маркеров эффективности.

**Результаты:**

Несмотря на то что в 2018 году было пролечено на 8,5% больше пациентов (5370 пациентов), чем в 2017 году (4948 пациентов), было использовано меньше на 140 ампул вакцины против бешенства и на 436 ампул антирабического иммуноглобулина, что позволило сэкономить 4202 доллара США. Из 56 пациентов, укушенных явно бешеными собаками, 50 были живы в течение 6 месяцев наблюдения. С остальными шестью пациентами связаться не удалось, но они не обратились ни в одну больницу с бешенством.

**Вывод:**

Новая схема оказалась более экономичной, чем четырехнедельный режим с введением вакцины в 2 участка тела, и настолько же эффективной. Эта схема рекомендуется для профилактики бешенства в странах, где заболевание является широко распространенным при дефиците вакцины против бешенства и иммуноглобулина.

## Introduction

Globally, rabies kills approximately 59 000 people annually, with the greatest disease burden falling upon the poorest regions, where few dogs are vaccinated and access to post-exposure prophylaxis is very limited.^1^^,^^2^ Although many mammals transmit rabies, 99% of disease in low- and middle-income countries is transmitted by unvaccinated dogs.^3^^,^^4^

Rabies is an acute, progressive encephalitis caused by *Rabies lyssavirus*.^5^^–^^8^ The disease is fatal if not treated effectively soon after a bite from a rabid dog, whose saliva contains rabies virions. Once inoculated into the skin or mucous membranes, the virus travels gradually along nerve axons towards the central nervous system and results in acute, progressive encephalitis.^6^^,^^9^ The average incubation period from being bitten to the appearance of symptoms is 6 weeks. Classically, hydrophobia and aerophobia develop and death follows from neuronal and autonomic dysfunction.^6^^,^^9^

In low- and middle-income countries, free-roaming dogs pose a danger both to people, especially children, and animals: financial loss can result from the death of livestock.^4^ Unfortunately many health-care providers have not kept pace with new developments in post-exposure prophylaxis, and unsafe home remedies in rural areas in many low- and middle-income countries increase the risk of death. Several studies found that some emergency department physicians in these countries were unaware of recent changes in rabies vaccine administration, whereas others were either afraid to infiltrate rabies immunoglobulin into wounds or considered it too expensive to be used.^10^^–^^14^ Moreover, many physicians did not even wash wounds thoroughly before referring victims to rabies prevention centres in distant towns or cities.

Human rabies prophylaxis has improved considerably since the nineteenth century.^15^^–^^17^ During the 1970s, the introduction of tissue culture vaccines created a gold standard for human rabies post-exposure prophylaxis, as we describe in the data repository.^18^ For many years, the five-dose, Thai Red Cross intradermal regimen was the preferred option for high-throughput clinics in low- and middle-income countries where rabies is endemic,^19^^,^^20^ whereas the intramuscular Essen regimen was prescribed in developed countries with few patients. Intradermal administration of rabies vaccine allowed smaller doses to be given without compromising efficacy or safety.^21^^–^^26^ Also, one ampoule could be shared among several patients, thereby decreasing the cost and improving vaccine availability in high-incidence areas. Researchers observed, however, that many patients skipped the final 90-day dose. Over the years, studies proved that missing this dose did not increase mortality. Thus, in 2006, the regimen was shortened to the updated two-site, 4-week, Thai Red Cross, intradermal schedule, which was applied in several countries where it mitigated vaccine supply shortages and reduced costs by 60–80%.^24^^,^^25^^,^^27^

In 2012, researchers showed that a two-site, 1-week, intradermal schedule was associated with a robust antibody response for up to 180 days in healthy volunteers and suggested further studies on dog bite victims.^28^^–^^30^ During 2017, in response to evidence from several studies that the fourth dose of the Thai Red Cross, intradermal vaccine schedule given on day 28 offered no additional benefits, the World Health Organization (WHO) constituted a Strategic Advisory Group of Experts working group to consider further improvements in the dosing schedule that could make post-exposure prophylaxis even more affordable and convenient without compromising safety or efficacy.^31^ In 2018, based on discussions by the Strategic Advisory Group of Experts, WHO published a position paper on post-exposure prophylaxis that proposed a shorter two-site, 1-week, intradermal vaccine regimen.^30^^–^^32^

Another problem was that rabies immunoglobulin, an important biological agent for treating severe wounds, is costly and frequently in short supply. Previously, the recommendation was that rabies immunoglobulin be infiltrated into the wound as much as anatomically possible and that any remaining immunoglobulin be injected into distant muscle. This recommendation, too, has been revised to eliminate the intramuscular injection because evidence showed that injected immunoglobulin did not increase protection and that precious rabies immunoglobulin could be saved without compromising efficacy.^33^^–^^38^ Even before 2018, there was strong support for, “improved access to post-exposure prophylaxis (PEP) for exposed persons, as well as extended dog vaccination…. and clear, simplified PEP regimens utilizing modern WHO pre-qualified vaccines and, in case of category-III exposures, appropriate administration of rabies immunoglobulin (RIG).”^39^

The aim of our study was to compare the cost, effectiveness and treatment adherence of two rabies post-exposure prophylaxis regimens: (i) the two-site, 1-week, intradermal vaccine schedule recommended by WHO in 2018, with infiltration of rabies immunoglobulin into serious wounds only; and (ii) the two-site, 4-week, intradermal vaccine schedule, with infiltration of immunoglobulin into wounds and distant muscle.

## Methods

We performed an implementation study at The Indus Hospital in Karachi, Pakistan, a tertiary care hospital that provides free care to all. The Rabies Prevention Center at the hospital is a high-throughput clinic that operates around the clock, is run by trained nurses and receives dog bite victims from all over Karachi and other districts of Sindh and Balochistan provinces. 

At the Rabies Prevention Center, the severity of dog bite wounds is assessed in accordance with WHO definitions and all bite victims are provided with the full range of rabies post-exposure prophylaxis recommended by WHO.^9^ Rabies exposure is classified using three categories. Category I indicates no exposure to rabies and no post-exposure prophylaxis is required; category II indicates a moderate risk of exposure, which requires rabies vaccine only; and category III indicates a severe wound, which necessitates both rabies vaccine and rabies immunoglobulin. Wound lavage is performed for all bites (see data repository).^18^

### Treatment

Before 2018, all patients with dog bite wounds and category-II or –III rabies exposure were given intradermal injections of IndiRab*®* rabies vaccine (Bharat Biotech, Hyderabad, India) – a cell culture-derived, inactivated vaccine supplied in 0.5 mL reconstituted ampoules – on days 0, 3, 7 and 28 of a 1-month schedule. In patients with category-III exposure, wounds were infiltrated with equine rabies immunoglobulin (Equirab*®*, Bharat Serums and Vaccines Ltd, Mumbai, India) at a standard dose of 40 IU per kg body weight, with any remaining immunoglobulin injected into a muscle distant from the wound.

After the 2017 meeting of WHO’s Strategic Advisory Group of Experts, one study author, who took part in the working group on rabies vaccine and immunoglobulin, apprised management at The Indus Hospital about the amended rabies post-exposure prophylaxis schedule and provided supportive evidence from clinical trials. Subsequently, in January 2018, the new schedule was implemented after management approval (see data repository).^18^

First, we made sure hospital staff understood the new regimen and felt comfortable answering queries from patients. Staff members at the Rabies Prevention Center were already trained in intradermal vaccine administration and in infiltrating rabies immunoglobulin into wounds. We obtained feedback on their perceptions of the new regimen and, accordingly, placed information leaflets in the patient waiting areas to allay patients’ anxiety and to counter misinformation. In addition, we held information sessions on the new regimen for emergency department staff and hospital physicians.

From January 2018, the new post-exposure prophylaxis regimen for patients with category-II or –III rabies exposure comprised intradermal administration of 0.1 mL of vaccine at two sites (i.e. each deltoid muscle) on days 0, 3 and 7 of a 1-week schedule. As the Rabies Prevention Center functions on a 24-hour basis, each 0.5 mL ampoule was usually used up; if a small volume was left over, a hospital employee was vaccinated as pre-exposure prophylaxis. In addition, patients with category-III rabies exposure had immunoglobulin infiltrated into wounds only. The amount of immunoglobulin administered was calculated by body weight and, depending upon the number and severity of bites, an entire ampoule may have been needed. If the calculated volume of rabies immunoglobulin was less than the amount required to inject into wounds, it was diluted with normal saline before injection.^9^ If there were several small wounds, the calculated amount was drawn into two or three syringes and infiltrated into each wound. Any unused immunoglobulin was refrigerated for up to 12 hours and saved for the next category-III patient. Wastage of rabies vaccine and immunoglobulin was kept to a minimum and each ampoule was used to treat the maximum number of patients, which saved on cost and obviated the need for extra procurement of consumables.

### Outcomes

To assess the effectiveness of treatment, we investigated survival in patients who were bitten by an apparently rabid dog. As diagnostic evidence of rabies virus infection in dogs was not available, we accepted the affected community’s characterization of an apparently rabid dog as one that displayed highly aggressive behaviour and hypersalivation. In addition, if five or more people were bitten without provocation by the same dog on the same day, we assumed that the bites carried a high risk of rabies. Documented survival of anyone bitten in this way 6 months after being treated was taken as indirect evidence of the effectiveness of treatment. In 2017, 61 people were bitten by apparently rabid dogs compared with 56 in 2018.

Patients were followed up by monthly phone calls for 6 months even though the average incubation period of the virus is 6 weeks. If there was no response the first time, a second call was placed at a different time within 1 week of the first. On average, two calls were made per patient each month during the follow-up period of 6 months. If a patient did not respond for two consecutive months, the patient’s home was visited by a member of the team.

Data on patients treated between 1 January and 31 December in both 2017 and 2018 were obtained from the health management information system at The Indus Hospital. Information on the number of ampoules of rabies vaccine and immunoglobulin consumed was obtained from the pharmacy department at the hospital and details of costs were obtained from the finance and procurement department.

### Statistical analysis

We report descriptive statistics for all patients treated during 2017 and 2018 and compare the cost of rabies post-exposure prophylaxis in these 2 years. We also computed the difference in the number of ampoules of rabies vaccine and immunoglobulin consumed in these years. Costs are expressed in United States dollars (US$) and were derived using the exchange rate for 2018, during which US$ 1 equalled 130 Pakistani rupees. The cost of consumables, such as needles, swabs and supplies for wound washing, were the same in both years and had no implications for expenditure.

The study was submitted to an institutional review board registered with the Office for Human Research Protections at the United States Department of Health and Human Services (registration number IRB 00005148). As the study involved a chart review and data were analysed anonymously, it was declared exempt from review by the institutional review board.

## Results

In 2017, 4998 patients with dog bites were registered at The Indus Hospital. In 2018, the number was 5465, 9.3% (467/4998) more than in 2017. The demographic characteristics of these patients were similar in the 2 years (Table 1). In both years, more than 80% of people bitten were male and over 30% were younger than 15 years. Almost all patients presented with category-II or -III rabies exposure and were given rabies vaccine: 99.0% (4948/4998) in 2017 and 98.3% (5370/5465) in 2018. Patients with category-III exposure also received rabies immunoglobulin: 45.0% (2249/4998) in 2017 and 43.3% (2365/5465) in 2018. Category-I victims received neither vaccine nor immunoglobulin.

**Table 1:** Demographic characteristics, dog bite victims, The Indus Hospital, Karachi, Pakistan, 2017 and 2018

Patient characteristic	Year, no. (%)	
	2017 (*n* = 4998)	2018 (*n* = 5465)
**Male**	4323 (86.5)	4581 (83.8)
**Female**	675 (13.5)	884 (16.2)
**Age, years**		
0–5	224 (4.5)	271 (4.9)
6–15	1699 (34.0)	1522 (27.9)
> 15	3075 (61.5)	3672 (67.2)
**Rabies exposure category^a^**		
I	50 (1.0)	95 (1.7)
II	2699 (54.0)	2761 (50.6)
III	2249 (45.0)	2609 (47.7)

^a^ Category I indicates no exposure to rabies, category II indicates a moderate risk of exposure and category III indicates a severe dog bite wound.

The details of the cost analysis are shown in Table 2. In 2017, 1.45 ampoules of vaccine were consumed per patient over 4 weeks at a cost of US$ 5.65 per ampoule, equivalent to US$ 8.19 per patient. Overall, 7174.6 ampoules were used to treat 4948 patients in 2017 at a total cost of US$ 40 536.50. In 2018, 1.31 ampoules were used per patient and the cost per patient was US$ 7.40: 9.6% (US$ 0.79/US$ 8.19) less than in 2017. Overall, 7034.7 ampoules were used in 2018 to treat 5370 patients, which was 8.5% (422/4948) more patients than in 2017. Despite the increase in patients, the total cost of vaccine in 2018 was US$ 39 746.00 – US$ 790.50 less than in 2017.

**Table 2:** Cost of rabies treatment, The Indus Hospital, Karachi, Pakistan, 2017 and 2018

Treatment characteristic	Year		Difference between years
	2017^a^	2018^a^	
**Rabies vaccine**			
Patients treated, no.	4948	5370	+422
Ampoules consumed, no.	7174.6	7034.7	−139.9
Ampoules consumed per patient, no.	1.45	1.31	−0.14
Cost per ampoule, US$^b^	5.65	5.65	0.00
Cost per patient, US$	8.19	7.40	−0.79
Total vaccine costs, US$	40 536.50	39 746.00	−790.50
**Rabies immunoglobulin**			
Patients treated, no.	2249	2365^c^	+116
Ampoules consumed, no.	2431	1995	−436
Ampoules consumed per patient, no.	1.08	0.84	−0.24
Cost per ampoule, US$	7.825	7.825	0.00
Cost per patient, US$	8.45	6.60	−1.85
Total immunoglobulin costs, US$	19 022.60	15 610.90	−3411.70
**Total vaccine and immunoglobulin costs, US$**	59 559.10	55 356.90	−4202.20

US$: United States dollar.

^a^ In 2017, patients received intradermal rabies vaccine over 1 month plus rabies immunoglobulin injections into wounds and muscle; in 2018, patients received intradermal rabies vaccine over 1 week plus rabies immunoglobulin injections into wounds only.

^b^ Pakistani rupees were converted into United States dollars (US$) at the 2018 conversion rate of US$ 1 to 130 Pakistani rupees.

^c^ Of 2609 patients with category-III rabies exposure, 244 did not receive immunoglobulin for the following reasons: (i) the dog that bit them was vaccinated or was kept under observation for 10 days; (ii) the patient had previously received rabies vaccine; (iii) the patient presented > 1 week after receiving rabies vaccine elsewhere; or (iv) the patient presented several weeks or months after the dog bite and the dog was still alive.

In 2017, all bite wounds in patients with category-III exposure were infiltrated with rabies immunoglobulin and the remaining volume was injected into muscle. Overall, 2431 ampoules of immunoglobulin were used that year to treat 2249 patients at a total cost of US$ 19 022.60. In 2018, despite the fact that the number of patients who received rabies immunoglobulin increased to 2365, an increase of 5.2% (116/2249), 436 fewer ampoules were consumed and the total cost was US$ 15 610.90. Hence, more patients were treated for US$ 3411.70 less. Overall, had we continued with the 2017 regimen in accordance with WHO 2005 guidelines,^40^ we would have used 612 more vaccine ampoules and 559 more rabies immunoglobulin ampoules in 2018 than with the new regimen. However, we were able to treat more patients in 2018 for US$ 4202.20 less.

In 2017, 71.8% (3587/4998) of patients completed the vaccine course over 1 month. In 2018, 78.5% (4290/5465) of patients completed the course over 1 week, which suggests that adherence to the shorter schedule was better. In 2018, there were six occasions reported to The Indus Hospital on which five or more people were bitten without provocation by the same dog on the same day and the dog was assumed to be rabid. Of the 56 patients bitten, 50 (89%) were alive at follow-up. The remaining six (11%) could not be contacted because they and their families had moved out of the locality. However, as none of the six patients presented to The Indus Hospital or any other hospital with signs of rabies during follow-up, we felt confident in assuming they were alive.

## Discussion

Timely and complete treatment of people bitten by rabid animals is important for reducing mortality due to the rabies virus. In countries with scarce resources and a chronic shortage of vaccine, shorter treatment protocols will save money and enable more people to be treated. In our study, we found that treatment with the abridged, three-dose, 1-week version of the previously updated Thai Red Cross regimen, which was recommended by WHO in 2018, was effective and associated with lower costs and good treatment adherence.

In 2019, researchers demonstrated through mathematical modelling that the abridged regimen can be used to treat more patients at less cost.^41^ In our study, we found evidence that this new regimen is effective. Moreover, compared with the previous 1-month regimen with immunoglobulin injections into wound and muscle, use of the abridged, two-site, 1-week regimen with dose-sparing immunoglobulin injections was associated with an annual cost saving despite an increase in patient volume.

We followed patients bitten by apparently rabid dogs (i.e. dogs that bit five or more people on a single day without provocation) for at least 6 months, which was similar to the follow-up period in a study by the Institut Pasteur in Cambodia that provided the groundwork for WHO’s 2018 recommendations.^36^ Overall, 89% of these patients were confirmed as being well and there were no reports of rabies-suspected deaths among the remaining 11%. Our findings also concur with the results of the RESIST-2 cohort study, which showed that the administration of rabies vaccine beyond day 7 offered no additional benefit.^41^^,^^42^ In addition, a clinical trial in Thailand established that a two-site, abridged regimen was noninferior in effectiveness and safety to a four-site, 1-week regimen.^29^ Hence, we are confident that the 2018 regimen is effective.

We were encouraged to continue with the rabies post-exposure prophylaxis regimen recommended by WHO in 2018 by our finding that it was effective and economical. This approach helped mitigate rabies vaccine and immunoglobulin shortages at The Indus Hospital. Moreover, patients did not have to travel to the Rabies Prevention Center to receive a vaccine dose on day 28, which not only saved their time and expense but also conserved time for hospital personnel, reduced overcrowding and economized on consumables, such as syringes and swabs. In Pakistan, where an estimated 40% of the population lives below the poverty line and a family of five may have an income of US$ 1.8 per day,^43^ people cannot afford to travel from remote areas on numerous occasions (see data repository).^18^ Hence, relieving them of even one visit can result in substantial savings to their meagre incomes.

The successful use of the shortened regimen at the Rabies Prevention Center was not without challenges. We presented our study data and experience at different local forums and, although we showed the shortened regimen to be noninferior and more cost-effective than previous regimens, we got a mixed response. Health-care providers were initially reluctant to accept the modified regimen because of concerns over efficacy. Moreover, some were worried about the lack of training on intradermal administration and others expressed reservations about patient safety.

Engaging key stakeholders and building confidence among health-care providers was key to overcoming this initial resistance and to dispelling scepticism. We achieved this by organizing 3-day workshops for small groups twice a year (which expanded to a bimonthly schedule) intended to introduce trainees to the new schedule and to share experience, including analyses of patient outcomes and patients’ adherence to the new regimen (see data repository).^18^ We used didactic lectures and instructional videos in local languages to impart practical training on history-taking, washing wounds, proper vaccine dilution, intradermal inoculation and wound injection techniques for rabies immunoglobulin. Since 2018, we have organized over 20 workshops in Karachi and other areas across Pakistan, including Badin in Sindh province, Quetta in Balochistan province and Abbottabad and Mansehra in Khyber Pakhtunkhwa province. In total, we have trained over 150 health-care providers in the abridged regimen and we intend to continue with refresher training. Coverage by mass media led to our efforts being recognized by the Sindh Health Department and we are now training providers in the use of the new regimen across Sindh province.

A limitation of our study was the absence of laboratory diagnostic facilities to confirm rabies infections in dogs. Consequently, we had to rely on indirect evidence that a person had been bitten by a rabid dog based on its behaviour and unprovoked biting of several people. In countries with a high disease burden, increasing rabies diagnostic capability is needed.

Globally, many people are at risk for dog attacks. Safe, affordable and effective biological agents are now available to prevent rabies. We found the abridged regimen, recommend by WHO in 2018, is cost-effective and noninferior to the 28-day regimen. We suggest the abridged regimen’s implementation in low- and middle-income countries where there is a high patient volume and erratic access to rabies vaccine and immunoglobulin. In the long run, rabies could be eliminated by educating the public, by risk assessment and by implementing the One Health approach through mass dog vaccination.^44^ Together, these strategies can help achieve the global vision of zero human deaths due to rabid dogs by 2030.^45^^–^^49^

## References

[1] Hampson K, Coudeville L, Lembo T, Sambo M, Kieffer A, Attlan M, et al.; Global Alliance for Rabies Control Partners for Rabies Prevention. Correction: Estimating the global burden of endemic canine rabies. PLoS Negl Trop Dis. 2015 5 11;9(5):e0003786. 10.1371/journal.pntd.0003786 25961848 PMC4427501

[2] Cleaveland S, Fèvre EM, Kaare M, Coleman PG. Estimating human rabies mortality in the United Republic of Tanzania from dog bite injuries. Bull World Health Organ. 2002;80(4):304–10. 12075367 PMC2567765

[3] Hossain M, Ahmed K, Bulbul T, Hossain S, Rahman A, Biswas MN, et al. Human rabies in rural Bangladesh. Epidemiol Infect. 2012 11;140(11):1964–71. 10.1017/S095026881100272X 22185694

[4] Widyastuti MD, Bardosh KL, Sunandar, Basri C, Basuno E, Jatikusumah A, et al. On dogs, people, and a rabies epidemic: results from a sociocultural study in Bali, Indonesia. Infect Dis Poverty. 2015 6 30;4(1):30. 10.1186/s40249-015-0061-1 26137295 PMC4486702

[5] Amarasinghe GK, Aréchiga Ceballos NG, Banyard AC, Basler CF, Bavari S, Bennett AJ, et al. Taxonomy of the order *Mononegavirales*: update 2018. Arch Virol. 2018 8;163(8):2283–94. 10.1007/s00705-018-3814-x 29637429 PMC6076851

[6] Burton EC, Burns DK, Opatowsky MJ, El-Feky WH, Fischbach B, Melton L, et al. Rabies encephalomyelitis: clinical, neuroradiological, and pathological findings in 4 transplant recipients. Arch Neurol. 2005 6;62(6):873–82. 10.1001/archneur.62.6.873 15956158

[7] Fu ZF, Jackson AC. Neuronal dysfunction and death in rabies virus infection. J Neurovirol. 2005 2;11(1):101–6. 10.1080/13550280590900445 15804968

[8] Rupprecht CE, Salahuddin N. Current status of human rabies prevention: remaining barriers to global biologics accessibility and disease elimination. Expert Rev Vaccines. 2019 6;18(6):629–40. 10.1080/14760584.2019.1627205 31159618

[9] WHO expert consultation on rabies: third report. WHO Technical Report Series, no. 1012. Geneva: World Health Organization; 2018. Available from: https://apps.who.int/iris/handle/10665/272364 [cited 2018 Apr 18].

[10] Salahuddin N, Mubashar K, Ansari B. Utilization of rabies immunoglobulin in seven urban Pakistan emergency rooms. Asian Biomed. 2013;7(2):243–7.

[11] Barbosa Costa G, Gilbert A, Monroe B, Blanton J, Ngam S, Recuenco S, et al. The influence of poverty and rabies knowledge on healthcare seeking behaviors and dog ownership, Cameroon. PLoS One. 2018 6 21;13(6):e0197330. 10.1371/journal.pone.0197330 29927935 PMC6013156

[12] Salahuddin N, Jamali S, Ibraheem K, Sardar S. Awareness about rabies post exposure prophylaxis in Pakistan among patients and health care workers: results from an Asian Rabies Expert Bureau study. J Coll Physicians Surg Pak. 2011 8;21(8):491–4. 21798137

[13] Tiwari HK, Vanak AT, O’Dea M, Robertson ID. Knowledge, attitudes and practices towards dog-bite related rabies in para-medical staff at rural primary health centres in Baramati, western India. PLoS One. 2018 11 16;13(11):e0207025. 10.1371/journal.pone.0207025 30444871 PMC6239288

[14] Zaidi SMA, Labrique AB, Khowaja S, Lotia-Farrukh I, Irani J, Salahuddin N, et al. Geographic variation in access to dog-bite care in Pakistan and risk of dog-bite exposure in Karachi: prospective surveillance using a low-cost mobile phone system. PLoS Negl Trop Dis. 2013 12 12;7(12):e2574. 10.1371/journal.pntd.0002574 24349590 PMC3861251

[15] Rupprecht CE, Gibbons RV. Clinical practice. Prophylaxis against rabies. N Engl J Med. 2004 12 16;351(25):2626–35. 10.1056/NEJMcp042140 15602023

[16] Warrell MJ. Developments in human rabies prophylaxis. Rev Sci Tech. 2018 8;37(2):629–47. 10.20506/rst.37.2.2829 30747121

[17] Wu X, Smith TG, Rupprecht CE. From brain passage to cell adaptation: the road of human rabies vaccine development. Expert Rev Vaccines. 2011 11;10(11):1597–608. 10.1586/erv.11.140 22043958

[18] Salahuddin N, Ansari N, Gohar A. One-week PEP Implementation & Operational Research.doc. Saving cost and more human lives with one-week rabies post-exposure prophylaxis in a high-throughput rabies prevention center in Karachi, Pakistan. Supplementary material. London: Figshare; 2021. 10.6084/m9.figshare.13547654.v6 10.6084/m9.figshare.13547654.v6

[19] Li AJ, Sreenivasan N, Siddiqi UR, Tahmina S, Penjor K, Sovann L, et al. Descriptive assessment of rabies post-exposure prophylaxis procurement, distribution, monitoring, and reporting in four Asian countries: Bangladesh, Bhutan, Cambodia, and Sri Lanka, 2017–2018. Vaccine. 2019 10 3;37 Suppl 1:A14–9. 10.1016/j.vaccine.2018.10.011 30314908 PMC6702106

[20] Khomvilai S, Daviratanasilpa S, Pornmuttakun D, Sakolpap L, Akesowan S, Pakmanee N, et al. Chapter twenty-four: Production of equine rabies immune globulin of high purity, potency, and safety. In: Rupprecht C, Nagarajan T, editors. Current laboratory techniques in rabies diagnosis, research and prevention. Volume 2. San Diego: Academic Press; 2015: 293–303. 10.1016/B978-0-12-801919-1.00024-5

[21] Quiambao BP, Dimaano EM, Ambas C, Davis R, Banzhoff A, Malerczyk C. Reducing the cost of post-exposure rabies prophylaxis: efficacy of 0.1 ml PCEC rabies vaccine administered intradermally using the Thai Red Cross post-exposure regimen in patients severely exposed to laboratory-confirmed rabid animals. Vaccine. 2005 2 25;23(14):1709–14. 10.1016/j.vaccine.2004.09.027 15705476

[22] Khawplod P, Wilde H, Sirikwin S, Benjawongkulchai M, Limusanno S, Jaijaroensab W, et al. Revision of the Thai Red Cross intradermal rabies post-exposure regimen by eliminating the 90-day booster injection. Vaccine. 2006 4 12;24(16):3084–6. 10.1016/j.vaccine.2006.01.051 16494972

[23] Undurraga EA, Meltzer MI, Tran CH, Atkins CY, Etheart MD, Millien MF, et al. Cost-effectiveness evaluation of a novel integrated bite case management program for the control of human rabies, Haiti 2014–2015. Am J Trop Med Hyg. 2017 6;96(6):1307–17. 10.4269/ajtmh.16-0785 28719253 PMC5462564

[24] Kundu BK, Meshram GG, Bhargava S, Meena O. Cost savings of using updated Thai Red Cross intradermal regimen in a high-throughput anti-rabies clinic in New Delhi, India. Trop Med Infect Dis. 2019 3 22;4(1):50. 10.3390/tropicalmed4010050 30909481 PMC6473397

[25] Quiambao BP, Ambas C, Diego S, Bosch Castells V, Korejwo J, Petit C, et al. Intradermal post-exposure rabies vaccination with purified Vero cell rabies vaccine: comparison of a one-week, 4-site regimen versus updated Thai Red Cross regimen in a randomized non-inferiority trial in the Philippines. Vaccine. 2019 4 10;37(16):2268–77. 10.1016/j.vaccine.2019.02.083 30890382

[26] Rupprecht CE, Briggs D, Brown CM, Franka R, Katz SL, Kerr HD, et al. Evidence for a 4-dose vaccine schedule for human rabies post-exposure prophylaxis in previously non-vaccinated individuals. Vaccine. 2009 11 27;27(51):7141–8. 10.1016/j.vaccine.2009.09.029 19925944

[27] Salahuddin N, Gohar MA, Baig-Ansari N. Reducing cost of rabies post exposure prophylaxis: experience of a tertiary care hospital in Pakistan. PLoS Negl Trop Dis. 2016 2 26;10(2):e0004448. 10.1371/journal.pntd.0004448 26919606 PMC4769324

[28] Sudarshan MK, Narayana DH, Madhusudana SN, Holla R, Ashwin BY, Gangaboraiah B, et al. Evaluation of a one week intradermal regimen for rabies post-exposure prophylaxis: results of a randomized, open label, active-controlled trial in healthy adult volunteers in India. Hum Vaccin Immunother. 2012 8;8(8):1077–81. 10.4161/hv.20471 22699446 PMC3551879

[29] Kerdpanich P, Chanthavanich P, De Los Reyes MR, Lim J, Yu D, Ama MC, et al. Shortening intradermal rabies post-exposure prophylaxis regimens to 1 week: results from a phase III clinical trial in children, adolescents and adults. PLoS Negl Trop Dis. 2018 6 6;12(6):e0006340. 10.1371/journal.pntd.0006340 29874228 PMC6005579

[30] Kessels J, Tarantola A, Salahuddin N, Blumberg L, Knopf L. Rabies post-exposure prophylaxis: a systematic review on abridged vaccination schedules and the effect of changing administration routes during a single course. Vaccine. 2019 10 3;37 Suppl 1:A107–17. 10.1016/j.vaccine.2019.01.041 30737043

[31] Rabies vaccines: WHO position paper – April 2018. Wkly Epidemiol Rec. 2018 4 20;93(16):201–20. Available from https://www.who.int/rabies/resources/who_wer9316/en/ [cited 2018 Apr 20]

[32] Tarantola A, Ly S, Chan M, In S, Peng Y, Hing C, et al. Intradermal rabies post-exposure prophylaxis can be abridged with no measurable impact on clinical outcome in Cambodia, 2003-2014. Vaccine. 2019 10 3;37 Suppl 1:A118–27. 10.1016/j.vaccine.2018.10.054 30454946

[33] Saesow N, Chaiwatanarat T, Mitmoonpitak C, Wilde H. Diffusion and fate of intramuscularly injected human rabies immune globulin. Acta Trop. 2000 10 2;76(3):289–92. 10.1016/S0001-706X(00)00107-8 10974171

[34] Bharti OK, Thakur B, Rao R. Wound-only injection of rabies immunoglobulin (RIG) saves lives and costs less than a dollar per patient by “pooling strategy”. Vaccine. 2019 10 3;37 Suppl 1:A128–31. 10.1016/j.vaccine.2019.07.087 31395454

[35] Tarantola A, Ly S, In S, Ong S, Peng Y, Heng N, et al. Rabies vaccine and rabies immunoglobulin in Cambodia: use and obstacles to use. J Travel Med. 2015 Sep-Oct;22(5):348–52. 10.1111/jtm.12228 26173470

[36] Tarantola A, Tejiokem MC, Briggs DJ. Evaluating new rabies post-exposure prophylaxis (PEP) regimens or vaccines. Vaccine. 2019 10 3;37 Suppl 1:A88–93. 10.1016/j.vaccine.2018.10.103 30471958

[37] Taylor E, Banyard AC, Bourhy H, Cliquet F, Ertl H, Fehlner-Gardiner C, et al. Avoiding preventable deaths: the scourge of counterfeit rabies vaccines. Vaccine. 2019 4 17;37(17):2285–7. 10.1016/j.vaccine.2019.03.037 30922698

[38] Soentjens P, Andries P, Aerssens A, Tsoumanis A, Ravinetto R, Heuninckx W, et al. Preexposure intradermal rabies vaccination: a noninferiority trial in healthy adults on shortening the vaccination schedule from 28 to 7 days. Clin Infect Dis. 2019 2 1;68(4):607–14. 10.1093/cid/ciy513 29939243

[39] Dodet B; Asian Rabies Expert Bureau (AREB). Report of the sixth AREB meeting, Manila, The Philippines, 10-12 November 2009. Vaccine. 2010 4 26;28(19):3265–8. 10.1016/j.vaccine.2010.02.093 20211220

[40] WHO expert consultation on rabies: first report. WHO Technical Report Series, no. 931. Geneva: World Health Organization; 2005. Available from: https://apps.who.int/iris/handle/10665/43262 [cited 2018 Apr 18]. 16485446

[41] Hampson K, Abela-Ridder B, Bharti O, Knopf L, Léchenne M, Mindekem R, et al. Modelling to inform prophylaxis regimens to prevent human rabies. Vaccine. 2019 10 3;37 Suppl 1:A166–73. 10.1016/j.vaccine.2018.11.010 30528846 PMC7612382

[42] Cantaert T, Borand L, Kergoat L, Leng C, Ung S, In S, et al. A 1-week intradermal dose-sparing regimen for rabies post-exposure prophylaxis (RESIST-2): an observational cohort study. Lancet Infect Dis. 2019 12;19(12):1355–62. 10.1016/S1473-3099(19)30311-1 31570311

[43] Nishtar S. Choked pipes: reforming Pakistan’s mixed health system. Oxford: Oxford University Press; 2010. 20419962

[44] Cleaveland S, Sharp J, Abela-Ridder B, Allan KJ, Buza J, Crump JA, et al. One Health contributions towards more effective and equitable approaches to health in low- and middle-income countries. Philos Trans R Soc Lond B Biol Sci. 2017 7 19;372(1725): 20160168. 10.1098/rstb.2016.0168 28584176 PMC5468693

[45] Gongal G, Wright AE. Human rabies in the WHO Southeast Asia Region: forward steps for elimination. Adv Prev Med. 2011;2011:383870. 10.4061/2011/383870 21991437 PMC3178116

[46] Kumarapeli V, Awerbuch-Friedlander T. Human rabies focusing on dog ecology: a challenge to public health in Sri Lanka. Acta Trop. 2009 10;112(1):33–7. 10.1016/j.actatropica.2009.06.009 19540826

[47] Lapiz SMD, Miranda MEG, Garcia RG, Daguro LI, Paman MD, Madrinan FP, et al. Implementation of an intersectoral program to eliminate human and canine rabies: the Bohol Rabies Prevention and Elimination Project. PLoS Negl Trop Dis. 2012;6(12):e1891. 10.1371/journal.pntd.0001891 23236525 PMC3516573

[48] Rupprecht CE, Bannazadeh Baghi H, Del Rio Vilas VJ, Gibson A D, Lohr F, Meslin FX, et al. Global rabies management: perspectives on regional strategies for prevention and control. Rev Sci Tech. 2018 8;37(2):711–27. 10.20506/rst.37.2.2835 30747114

[49] Welburn SC, Beange I, Ducrotoy MJ, Okello AL. The neglected zoonoses–the case for integrated control and advocacy. Clin Microbiol Infect. 2015 5;21(5):433–43. 10.1016/j.cmi.2015.04.011 25911990

